# Disrupting SOD1 activity inhibits cell growth and enhances lipid accumulation in nasopharyngeal carcinoma

**DOI:** 10.1186/s12964-018-0240-3

**Published:** 2018-06-11

**Authors:** Shuai Li, Lanyan Fu, Tian Tian, Liwen Deng, Huangbin Li, Weixiong Xia, Qing Gong

**Affiliations:** 1 Department of Biochemistry and Molecular Biology, GMU-GIBH Joint School of Life Sciences, Guangzhou Medical University, Guangzhou, 510182 People’s Republic of China; 2 State Key Laboratory of Oncology in South China, Collaborative Innovation Center for Cancer Medicine, Sun Yat-sen University Cancer Center, Guangzhou, 510060 People’s Republic of China

**Keywords:** SOD1, ROS, Nasopharyngeal carcinoma, CPT1A

## Abstract

**Background:**

SOD1 is an abundant enzyme that has been studied as a regulator of the antioxidant defence system, and this enzyme is well known for catalyzing the dismutation of superoxide into hydrogen peroxide. However the SOD1 in the progress of NPC and underlying mechanisms remain unclear.

**Methods:**

In NPC tissue samples, SOD1 protein levels were measured by Western blot and immunohistochemical (IHC) staining. mRNA levels and SOD1 activity were monitored by qRT-PCR and SOD activity kit, respectively. Kaplan-Meier survival analysis was performed to explore the relationship between SOD1 expression and prognosis of NPC. The biological effects of SOD1 were investigated both in vitro by CCK-8, clonogenicity and apoptosis assays and in vivo by a xenograft mice model. Western blotting, ROS assay and triglyceride assays were applied to investigate the underlying molecular mechanism of pro-survival role of SOD1 in NPC.

**Results:**

We observed a significant upregulation of SOD1 in NPC tissue and high SOD1 expression is a predictor of poor prognosis and is correlated with poor outcome. We confirmed the pro-survival role of SOD1 both in vitro and in vivo. We demonstrated that these mechanisms of SOD1 partly exist to maintain low levels of the superoxide anion and to avoid the accumulation of lipid droplets via enhanced CPT1A-mediated fatty acid oxidation.

**Conclusions:**

The results of this study indicate that SOD1 is a potential prognostic biomarker and a promising target for NPC therapy.

**Electronic supplementary material:**

The online version of this article (10.1186/s12964-018-0240-3) contains supplementary material, which is available to authorized users.

## Background

Altered redox status is a key biochemical feature that is frequently observed in tumour cells [1]. At moderate levels, reactive oxygen species (ROS) are important for tumourigenesis and tumour progression, but a large increase in the ROS level usually causes cell death [2, 3]. Superoxide is the first species to be produced, and superoxide is converted to hydrogen peroxide through dismutase activity [4]. The cellular detoxification of harmful superoxide usually requires the dismutase activity. Cancer cells are characterized by elevated levels of ROS, due to aberrant metabolism, cancer cells accumulate excessive ROS, thus requiring a robustly active antioxidant system to prevent cellular damage [5, 6]. Common ROS include superoxide (O_2_•-) and hydrogen peroxide (H_2_O_2_). Cellular ROS homeostasis is maintained by balancing the production of ROS with the activity of the antioxidant system. ROS-producing (i.e., ROS inducer) pathways and ROS-detoxifying (i.e., ROS scavenger) pathways are tightly regulated to avoid oxidative stress.

In eukaryotic cells, there are three distinct superoxide dismutases (SODs) [7]. SOD1 is a soluble Cu/Zn enzyme that is mainly located in the cytosol, although a small percentage of SOD1 proteins (~ 3%) is found in the intermembrane space of the mitochondria. SOD2 is a manganese-dependent enzyme located in the mitochondria, whereas SOD3 is an extracellular enzyme.

Nasopharyngeal carcinoma (NPC) is a commonly occurring cancer that has the highest incidence of malignant proliferation among head and neck cancers [8]. Our previous studies demonstrated that disrupting redox homeostasis enhances chemo sensitivity in colorectal cancer and prompts anoikis in NPC [6, 9, 10]. In this study, we aimed to assess the role of SOD1 in NPC cell growth. We provide evidence, both in vitro and in vivo, that restricting SOD1 function dampens the viability of NPC cells and increases superoxide (O_2_•-) production. Moreover, overexpression of SOD1 promotes the expression of CPT1A, while inhibits SOD1 decreases CPT1A and induces the accumulation of lipid droplets. Taken together, our study unravels a new mechanism showing that the SOD1/CPT1A axis is critical to support cancer cell growth.

## Methods

### Cells and reagents

All NPC cells were maintained in RPMI 1640 supplemented with 10% FBS. NP69 cells were maintained in serum-free keratinocyte medium supplemented with human recombinant epidermal growth factor (0.2 ng/mL) and bovine pituitary extract (20 μg/mL). SOD1 inhibitor LCS-1, CPT1a inhibitor Etomoxir, 4-Hydroxy-TEMPO (TEMPO), Phenazine methosulfate (PMS) was purchased from Sigma-Aldrich.

### Cell viability assays

Cells were plated at a density of 3000 cells per well in 96-well plates. Cell viability was measured using the CCK8 (Dojindo, Japan) assay according to the manufacturer’s protocol.

### Apoptosis assays

Apoptosis was assessed by Annexin-V/PI detection. Cells were harvested and a mesh screen method was used to prepare single-cell suspensions, which were stained per the manufacturer’s recommendation. Experiments were repeated three times.

### ROS assays

Dihydroethidium (Life Technologies) was added to each well at a concentration of 10 μM. After 30 min, the samples were analysed with a flow cytometer (20,000 events) or fluorescence microplate (Equal protein lysate) to detect the fluorescence. A Superoxide Assay Kit (Beyotime Institute of Biotechnology, Jiangsu, China) was also used to detect superoxide anionin accordance with the manufacturer’s instructions.

### SOD activity

SOD activity was measured using a Cu-Zn/MnSOD assay kit (WST) (Beyotime Institute of Biotechnology, Jiangsu, China). MnSOD activity was measured by adding 10 mM potassium cyanide to inactivate Cu-Zn/SOD activity. The difference between total SOD and MnSOD activity was considered the Cu-Zn/SOD activity. SOD activity was expressed as units/mg of protein (one unit was defined as the amount of enzyme that inhibited WST-1 reduction by approximately 50%).

### Lipid assays

Oil Red O and Nile Red are used for neutral lipid stains, cells were washed with PBS and were incubated with 1 ng/mL Nile red (Sigma-Aldrich) or 0.5% Oil red O (Sigma-Aldrich) solution for 10 min at room temperature. Cell nuclei were counterstained with DAPI (Sigma-Aldrich) or hematoxylin, and the cells were visualized under a fluorescence microscope. Intracellular triglycerides were assayed using a triglyceride assay kit (GPO-POD; Applygen Technologies, Inc., Beijing, China) according to the manufacturer’s recommended protocol.

### RNA isolation and quantitative real-time PCR

Total RNA isolation was carried out with TRIzol (Invitrogen) reagent following the manufacturer’s protocol. Total RNA (500 ng) was used for reverse transcription and quantitative real-time PCR analysis (qRT-PCR). The relative mRNA quantity was determined using the comparative cycle threshold (ΔΔCt) method. β-Actin expression was used for normalization. The primers used are listed in Additional file 1: Table S1.

### siRNA transfection and plasmids

For siRNA knockdown, SOD1 siRNA and a control siRNA were purchased from RiboBio. For SOD1 or CPT1A overexpression, pcDNA(3.1+)SOD1 or pcDNA(3.1+)CPT1A and a control vector were transfected according to the manufacturer’s instructions; transfections were performed using Lipofectamine 3000 (Qiagen) when cells were approximately 70% confluent. The sequences targeting SOD1 were as follows: 5’GCATGGATTCCATGTTCAT3′(#1), 5’CGTTTGGCTTGTGGTGTAA 3’(#2).

### Western blotting

For total protein extraction, cells were harvested and lysed. The protein concentration was determined using a BCA protein assay kit (Thermo Scientific) according to manufacturer’s protocol. Aliquots of equal amounts of cell lysate protein were subjected to Western blot analysis. Antibodies specific for SOD1 (Cell Signaling Technology), CPT1A (Proteintech), GAPDH (Cell Signaling Technology), ATGL (Proteintech), APGAT1 (Proteintech), DGAT1 (Proteintech), PDHE1A (Cell Signaling Technology), E-cadherin (Cell Signaling Technology), Vimentin (Cell Signaling Technology) and β-actin (Sigma) were used.

### Animal experiments

Female BABL/c athymic nude mice (4–5 weeks old) were obtained from the Guangdong Province Laboratory Animal Center (Guangzhou, China). Cells (1 × 10^6^) were injected subcutaneously into the flank of mice (5 mice/group). Every four days after injection, tumour sizes were measured using an unblinded manner as described previously [11]. To evaluate the anti-tumour effects of LCS-1, the mice were assigned to either the control or LCS-1 group (5 mice/group). The LCS-1 group received 0.76 mg/kg LCS-1 every four days for four weeks. After four weeks of treatment, the tumour volumes were examined, the mice were sacrificed, and the tumours were removed, embedded in paraffin and sectioned. All animal procedures were in accordance with the guidelines of the Institutional Animal Care and Use Committee.

### Patient information

The archived NPC paraffin sections (*n* = 100), NPC tissue specimens (*n* = 40) or nasopharynx epithelial tissues (NET; *n* = 11) were obtained from our hospital (Sun Yat-sen University Cancer Center (SYSUCC, Guangzhou, China). Clinicopathological parameter data were obtained from patient clinical records and pathological reports. The use of these clinical NPC specimens was approved by the Institutional Research Ethics Committee.

### Immunohistochemistry (IHC) and terminal-deoxynucleotidyl transferase mediated nick end labeling (TUNEL) analysis

Immunohistochemical analysis was conducted according to standard procedures described previously [10]. The prepared slides were incubated with antibodies against SOD1 (1:200 dilution). As a negative control, slides were incubated instead of primary antibody. The TUNEL assays were performed with the DeadEnd™ Colorimetric TUNEL System Kit (Promega, Cat. No.G3250) according to the manufacturer’s instructions.

### Statistical analyses

Data are presented as the means ± SD. Differences between the experimental groups were assessed by ANOVA or a two-tailed Student’s t-test. The log-rank test was used for survival analysis. Differences analyzed by GraphPad Prism 5 with *p* value less than 0.05 was considered statistically significant.

## Results

### High SOD1 expression is associated with poor prognosis in nasopharyngeal carcinoma

**Fig. 1:**
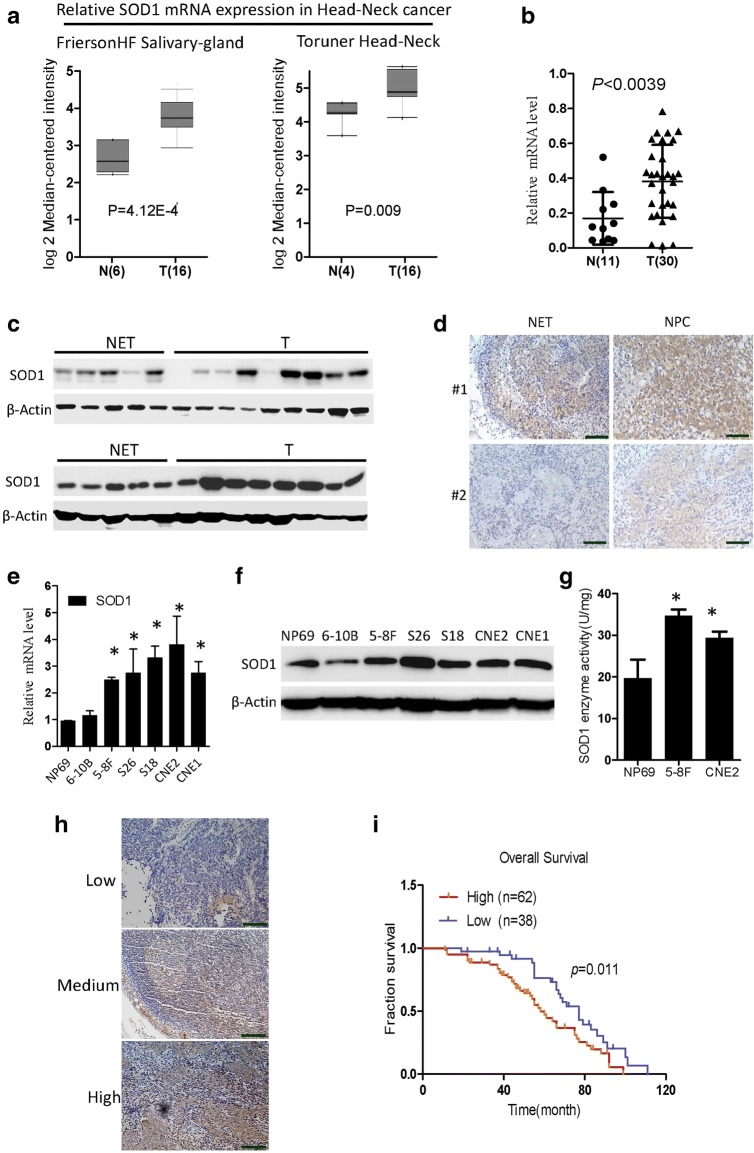
High SOD1 expression is associated with poor prognosis in NPC. **a** and **b** SOD1 is overexpressed in head and neck cancer (microarray data sets available from Oncomine (https://www.oncomine.com//)), and NPC tissues from the hospital (SYSUCC) were analysed by qPCR assays. **c** Immunoblotting analysis of SOD1 in ten NETs and NPC tissues (T). **d** Representative staining showing a relatively high expression of SOD1 in human NPC tissues compared with that in NET tissues, as analysed by IHC staining. **e** and **f** qPCR and immunoblotting analysis of SOD1 expression in nasopharynx epithelial cells and NPC cell lines; data are presented as the means ±SD (*n* = 3) *p < 0.05 for the indicated comparison (t-test). **g** SOD1 enzymatic activity in 5-8F and CNE2 cells was measured using an SOD Assay Kit-WST. **h** and **i** The overall survival and progression-free survival curves of patients with low and high SOD1 expression were generated using the Kaplan-Meier method and log-rank test

SOD1 is best known for its role in redox homeostasis and is often dysregulated during cancer development. We found that SOD1 mRNA was significantly increased in head and neck cancer tissues from the Oncomine microarray database (Fig. 1a, https://www.oncomine.org). Additionally, the SOD1 mRNA level was significantly increased in NPC tissues from our institute (SYSUCC) (Fig. 1b). The SOD1 protein level was notably increased in 17 representative tumours compared with normal tissues (Fig. 1c). We also analysed SOD1 expression in 100 human NPC tissue biopsies and 10 normal nasopharyngeal epithelial tissue biopsies. Representative IHC staining confirmed that, compared with that in normal tissues, SOD1 expression in human paraffin-embedded NPC tissues substantially increased (Fig. 1d). The expression profiles of SOD1 were also analysed in NPC cell lines, and compared with those in non-tumourigenic NP69 cells, both the mRNA and protein levels of SOD1 in NPC cells increased (Fig. 1e and f). As expected, compared with that in NP69 cells, the enzymatic activity of SOD1 in both 5-8F cells and CNE2 cells also increased (Fig. 1g). Together, these results suggest that SOD1 expression was significantly increased in human NPC cells and NPC tissues and that it may act as a key regulator of the antioxidant defence system in NPC.

To determine the clinical importance of this finding, we next evaluated the expression of SOD1 in 100 archived NPC tissues (Fig. 1h). Patients with low SOD1 expression had more favourable clinical outcomes, while patients with high SOD1 expression had shorter survival times (Fig. 1i). On the basis of the above observations, we concluded that low SOD1 protein expression predicts a favourable clinical outcome and better survival.

### SOD1 knockdown disrupts NPC cell growth in vitro

**Fig. 2:**
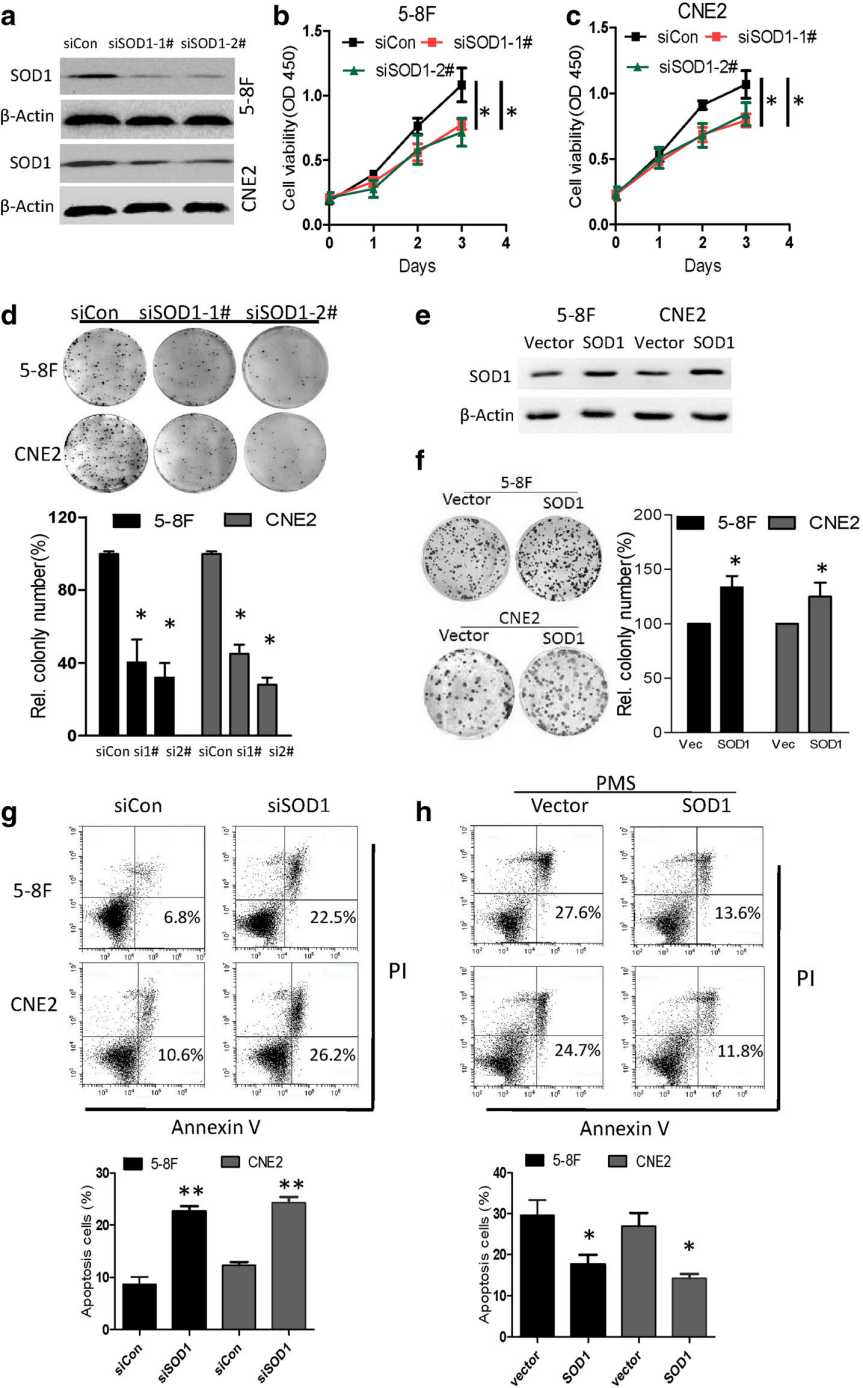
Knockdown of SOD1 expression in NPC cells inhibits cell viability and induces apoptosis. **a** Immunoblotting analysis of SOD1 in 5-8F and CNE2 cells transfected with two specific siRNAs or control siRNA. **b** and **c** Analysis of cell viability and clonogenicity in 5-8F and CNE2 cells with SOD1 knockdown. **d** Analysis clonogenicity in 5-8F and CNE2 cells with SOD1knocked down. **e** 5-8F and CNE2 cells were transfected with SOD1 plasmids and vector. **f** Clonogenicity of 5-8F and CNE2 cells with SOD1overexpression. **g** Cell apoptosis was measured by Annexin-V/PI assays in control cells and cells with SOD1 knockdown for 48 h. **h** Cell apoptosis was measured in SOD1-overexpressing and vector cells treated with PMS (25 μM) for 48 h. All error bars represent the S.D. of at least three replicates from two independent experiments.*p < 0.05, **p < 0.01 for the indicated comparison

Since SOD1 is overexpressed in NPC cancer cells, to examine the effect of SOD1 on NPC cells, SOD1 was knocked down using siRNA in 5-8F and CNE2 cells (Fig. 2a). Compared with the control cells, cells with SOD1 knocked down presented with reduced viability and clonogenicity (Fig. 2b-d). The overexpression of SOD1 (Fig. 2e) increased clonogenicity (Fig. 2f) and conferred resistance to apoptosis induced by the ROS inducer phenazine methosulfate (PMS) (Fig. 2h). Conversely, the knockdown of SOD1 increased the number of apoptotic cells (Fig. 2g).

### Knocking down SOD1 expression reduces NPC cell growth in vivo

**Fig. 3:**
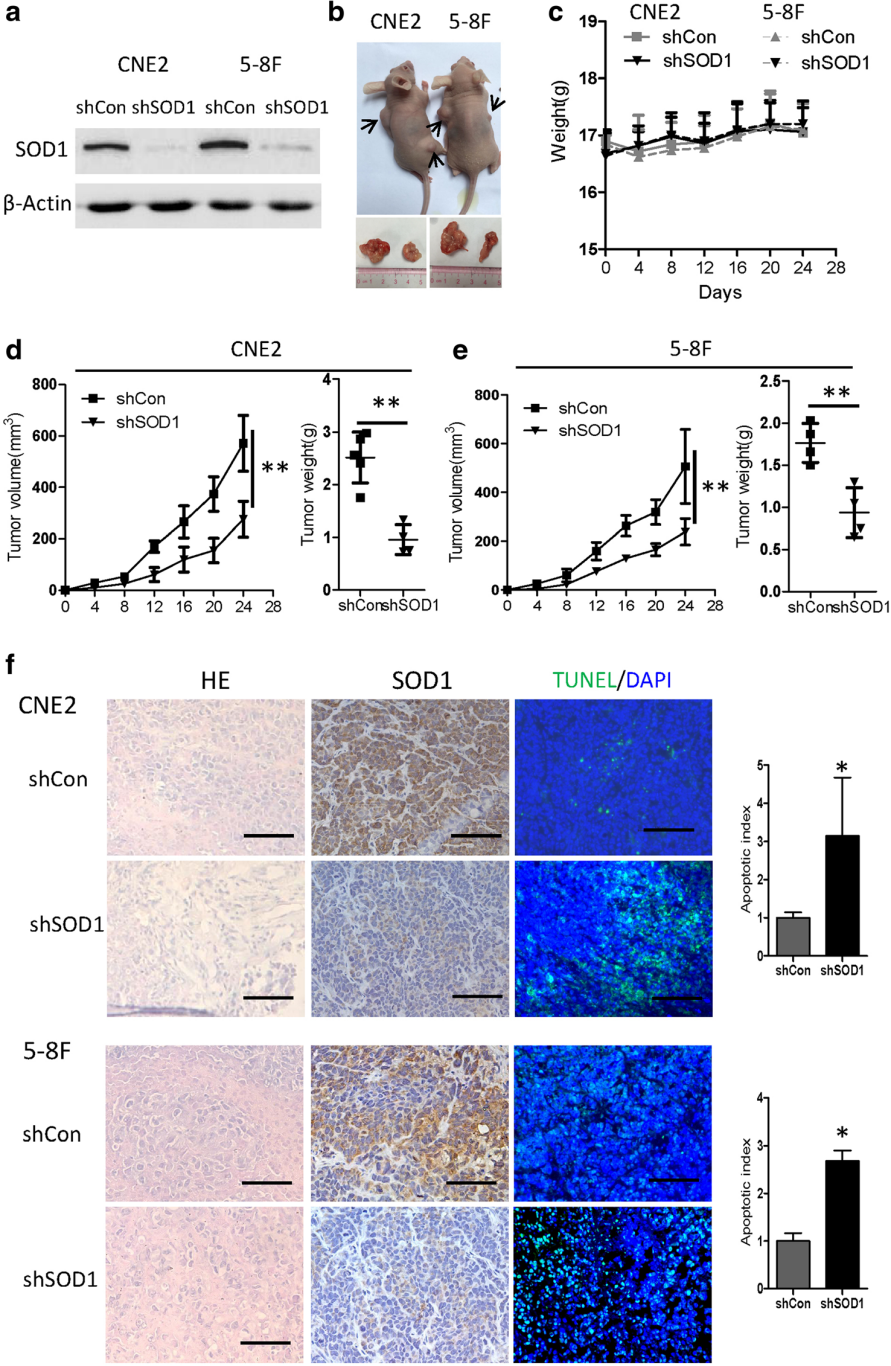
Knockdown of SOD1 suppresses NPC cell growth in vivo. **a** 5-8F and CNE2 cells were infected with Ad-shCon or Ad-shSOD1 adenovirus. At 72 h after infection, cell lysates were immunoblotted for SOD1. **b** The 5-8F and CNE2 cells were subcutaneously injected into the left (Ad-shCon, 1 × 10^6^/mouse) and right flank (Ad-shSOD1, 1 × 10^6^/mouse) of nude mice. Representative images of tumours derived from the indicated groups are shown. **c** The weight of the mice in the indicated groups was evaluated at each time point. **d** and **e** The tumour growth curves (left panel) and tumour weights (right panel) of the indicated group of mice were measured and recorded. **f** Paraffin-embedded tumour sections from mice with CNE2 and5-8F cell xenografts were stained with haematoxylin and eosin or SOD1 antibody. Apoptotic cells were visualized and quantified by TUNEL staining (green) and were counterstained with DAPI (blue).Scale bar: 100 μm. All error bars represent the S.D. of results from five mice.*p < 0.05, **p < 0.01, versus the corresponding control

Next, we further investigated whether SOD1 knockdown affects NPC cell growth in cell-based xenografts. Using a recombinant adeno associated virus, we first confirmed SOD1 knockdownin 5-8F and CNE2 cells (Fig. 3a). To evaluate the consequences of SOD1 loss in vivo, we used a nude mouse tumour model and administered 5-8F-shCon or 5-8F-shSOD1 cells and CNE2-shCon or CNE2-shSOD1 cells via subcutaneous injections into the flank. Compared with the vector control-derived tumours, the tumours from 5-8F or CNE2 cells with SOD1 knockdown presented with significant growth inhibition (Fig. 3b-e) and an increased number of apoptotic cells (Fig. 3f). These results suggest that disrupting SOD1 reduces NPC cell growth in vivo.

### LCS-1 suppresses NPC cell growth in vitro and in vivo

**Fig. 4:**
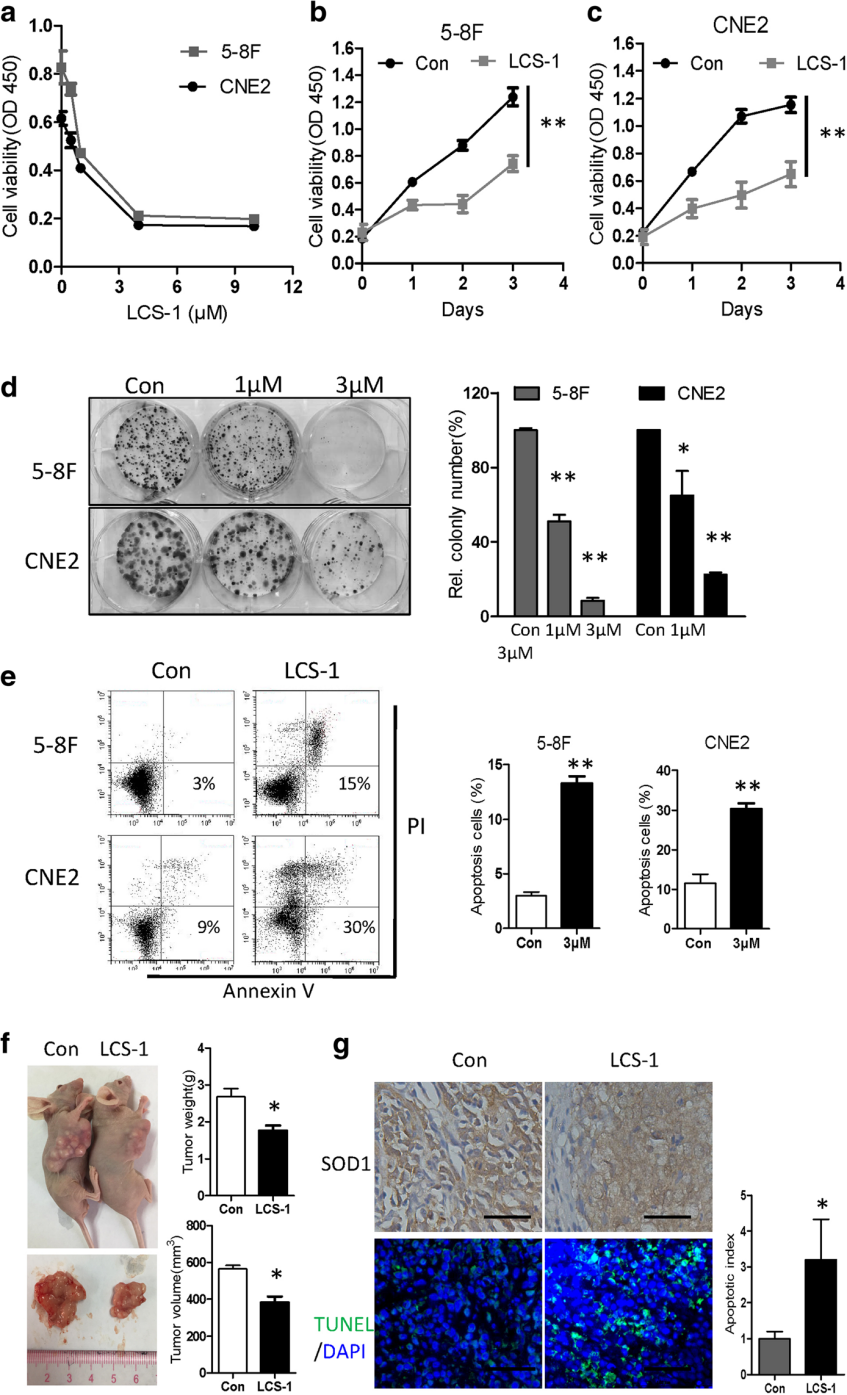
SOD1 suppression by LCS-1 induces apoptosis and inhibits proliferation. **a** The viability of the indicated cells exposed to LCS-1 (24 h) was detected with a CCK-8 kit. **b** and **c** CCK-8 assays of 5-8F and CNE2 cells treated with LCS-1 (1 μM) at the indicated time points. **d** Images (left panel) and quantification (right panel) of the clonogenicity of 5-8F and CNE2 cells treated with LCS-1. **e** Cell apoptosis was measured by Annexin-V/PI assays in LCS-1-treated cells and control cells for 48 h. **f** Paraffin-embedded tumour sections from mice with CNE2 cell xenografts were stained with haematoxylin and eosin or SOD1 antibody. Apoptotic cells were visualized and quantified by TUNEL staining (green) and were counterstained with DAPI (blue). Scale bar: 50 μm. All error bars represent the S.D. of at least three replicates from two independent experiments. *p < 0.05, **p < 0.01, versus the corresponding control

To further characterize the contribution of SOD1 to NPC cell growth, we treated 5-8F and CNE2 cells with the specific SOD1 inhibitor LCS-1. As shown in Fig. 4a-c, LCS-1 dose- and time-dependently inhibited the proliferation of 5-8F and CNE2 cells. Moreover, compared with the control-treated cells, 5-8F and CNE2 cells treated with LCS-1 had decreased clonogenicity and increased apoptosis (Fig. 4d and e). To further investigate whether LCS-1 affects NPC growth in vivo, CNE2 cells (1 × 10^6^) were injected subcutaneously into the mouse flank. Ten days later, LCS-1 (0.76 mg/kg) was administered every 4 days for 4 weeks. Compared with the control group, the LCS-1-treated group presented a greater reduction in tumour weight and growth (Fig. 4f) but a higher percentage of TUNEL-positive cells (Fig. 4g).

The above data showed that inhibiting SOD1 by LCS-1 treatment drastically reduced NPC cell viability in vitro.

### SOD1 suppression increases ROS and induces lipid accumulation

**Fig. 5:**
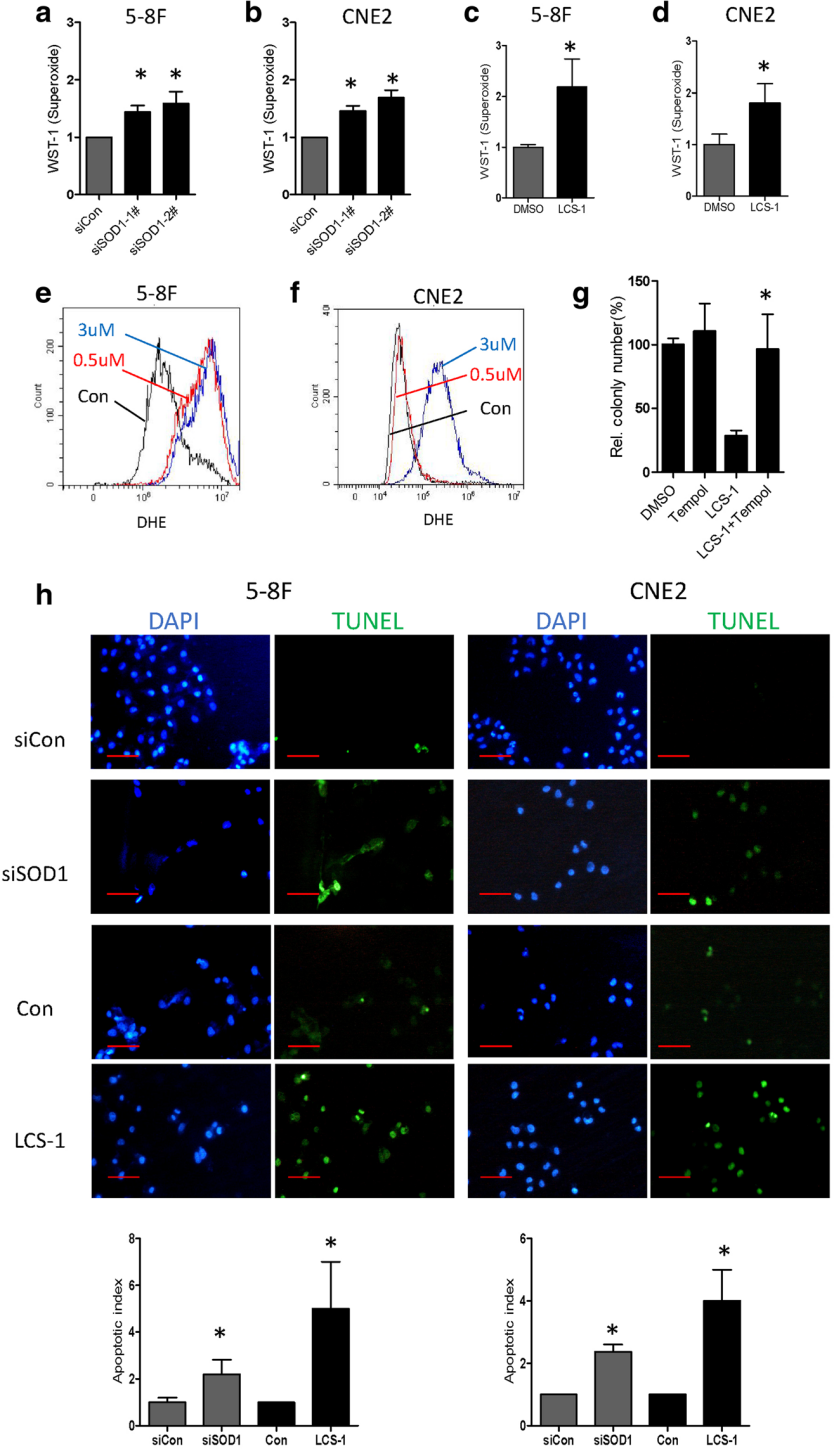
SOD1 suppression disrupts ROS homeostasis. **a** and **b** O_2_•- levels were measured by a superoxide kit in the indicated cells after treated by siRNAs for 24 h. **c** and **d** O_2_•- levels were measured by a superoxide kit in the indicated cells after treated by LCS-1 for 12 h. **e** and **f** Increased O_2_•- levels in the indicated cells treated with LCS-1 in comparison with those in the control cells, the flow cytometry analysis results are shown. **g** Quantification of clonogenicity of 5-8F cells in the indicated groups. **h** Apoptotic cells were visualized by TUNEL staining (green) and were counterstained with DAPI (blue). Scale bar: 100 μm. All error bars represent the S.D. of at least three replicates from two independent experiments.* *p* < 0.05 compared with the control

Because SOD1 acts as a regulator of anti-oxidative capacity, we posited that SOD1 knockdown may increase superoxide ion and induce cellular apoptosis. As expected, compared with the control cells, cells with SOD1 knocked down (Fig. 5a and b) or subjected to LCS-1 treatment (Fig. 5c-f) exhibited a substantial increase in superoxide levels, and LCS-1-inhibited clonogenicity was rescued by the administration of TEMPO, a superoxide dismutase mimetic (Fig. 5g). Furthermore, eliminating superoxide anion suppression by knocking down SOD1 or treating with LCS-1 caused a marked increase in apoptosis, as detected by the TUNEL assay (Fig. 5h).

These findings demonstrate that SOD1 inhibition increases cellular superoxide and induces apoptosis, which indicated that SOD1 is required for NPC cell detoxification.

**Fig. 6:**
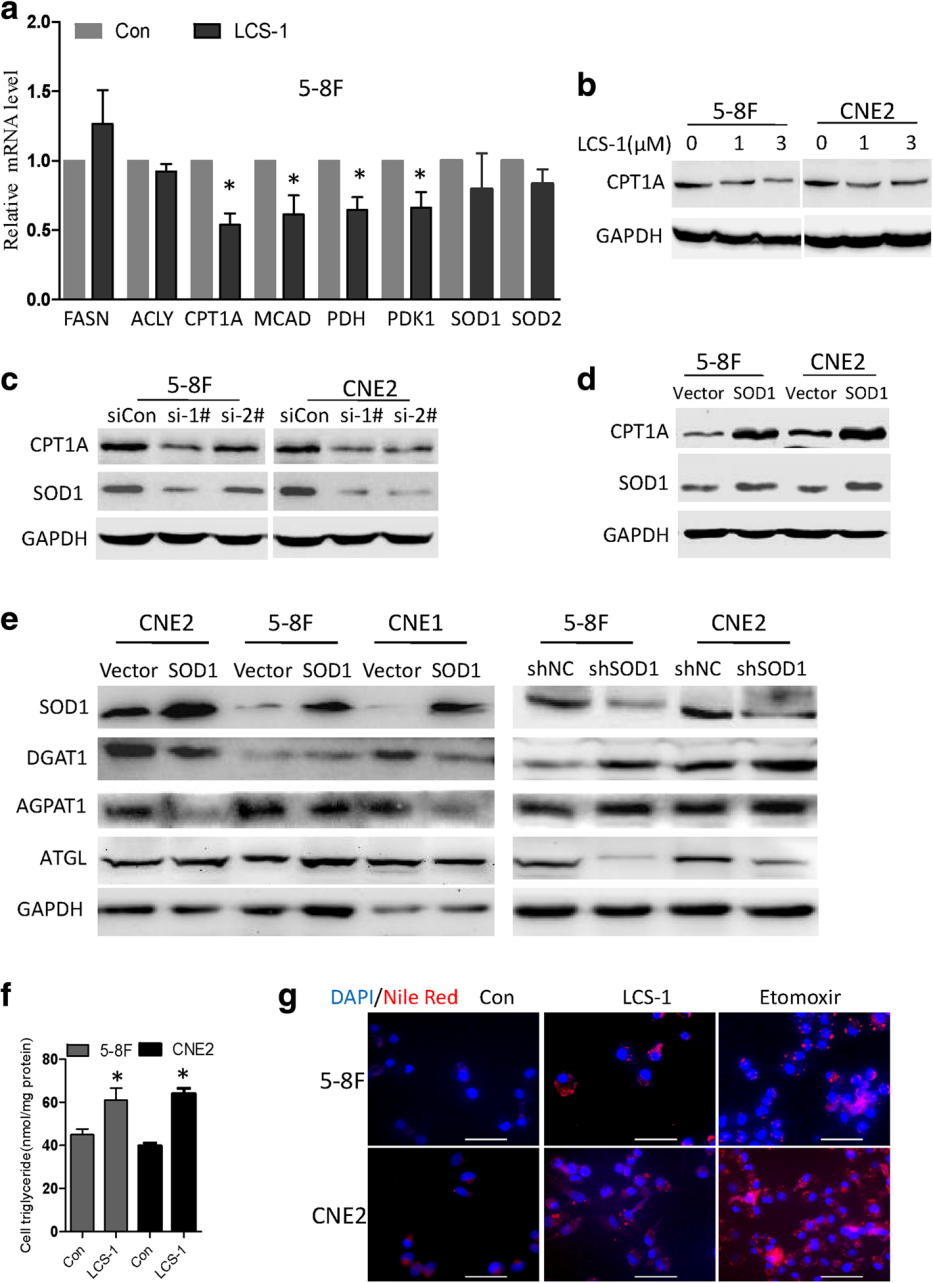
SOD1 suppression induces lipid accumulation. **a** Quantitative real-time PCR results of mitochondrial metabolism genes in 5-8F cells treated with LCS-1 (3 μM). **b** Immunoblotting analysis of CPT1A expression in 5-8F and CNE2 cells treated with LCS-1. **c** SOD1 knockdown by siRNA down-regulated CPT1A expression. **d** Immunoblotting analysis of CPT1A expression in 5-8F and CNE2 cells transfected with SOD1 or vector. **e** Western blot analysis of DGAT, AGPAT1 ATGL in 5-8F and CNE2 cells transfected with SOD1 plasmid and shRNA or control for 48 h. **f** Detection of the influence of LCS-1(3 μM) treatment on the triglyceride content by a triglyceride kit. **g** Nile red staining detection of the influence of LCS-1(3 μM) or etomoxir (400 μM) treatment on the lipid droplet. Scale bar: 50 μm. All error bars represent the S.D. of three replicates from two independent experiments. * p < 0.05 compared with the control

Recent studies have found that SOD1 not only functions as an antioxidant enzyme but also plays a key role in cellular metabolism [12–14]. The enhanced requirement of FAO for ATP and NADPH is frequently occurs in proliferating cells [15], block lipid oxidation via CPT1 as a therapeutic target for prostate cancer and myeloma [16, 17]. Inhibiting FA transport into the mitochondria is also able to effectively kill chronic lymphocytic leukaemia cells [18] and breast cancer [19]. Reducing fatty acid transport into the mitochondria forced fatty acids to lipid droplets for storage in Clear cell renal cell carcinoma [20]. To determine if SOD1 affects NPC cell mitochondrial metabolism, some of the key enzymes involved in mitochondrial pyruvic acid and FAO metabolism were examined. After 24 h, compared with the control cells, the LCS-1-treated 5-8F cells exhibited lower PDHE1A and CPT1A mRNA levels (Fig. 6a). Though SOD1 inhibition reduced PDHE1A mRNA expression, we observed minimal difference in the proliferation of siPDHE1A cells relative to control cells (Additional file 1: Figure S1).

**Fig. 7:**
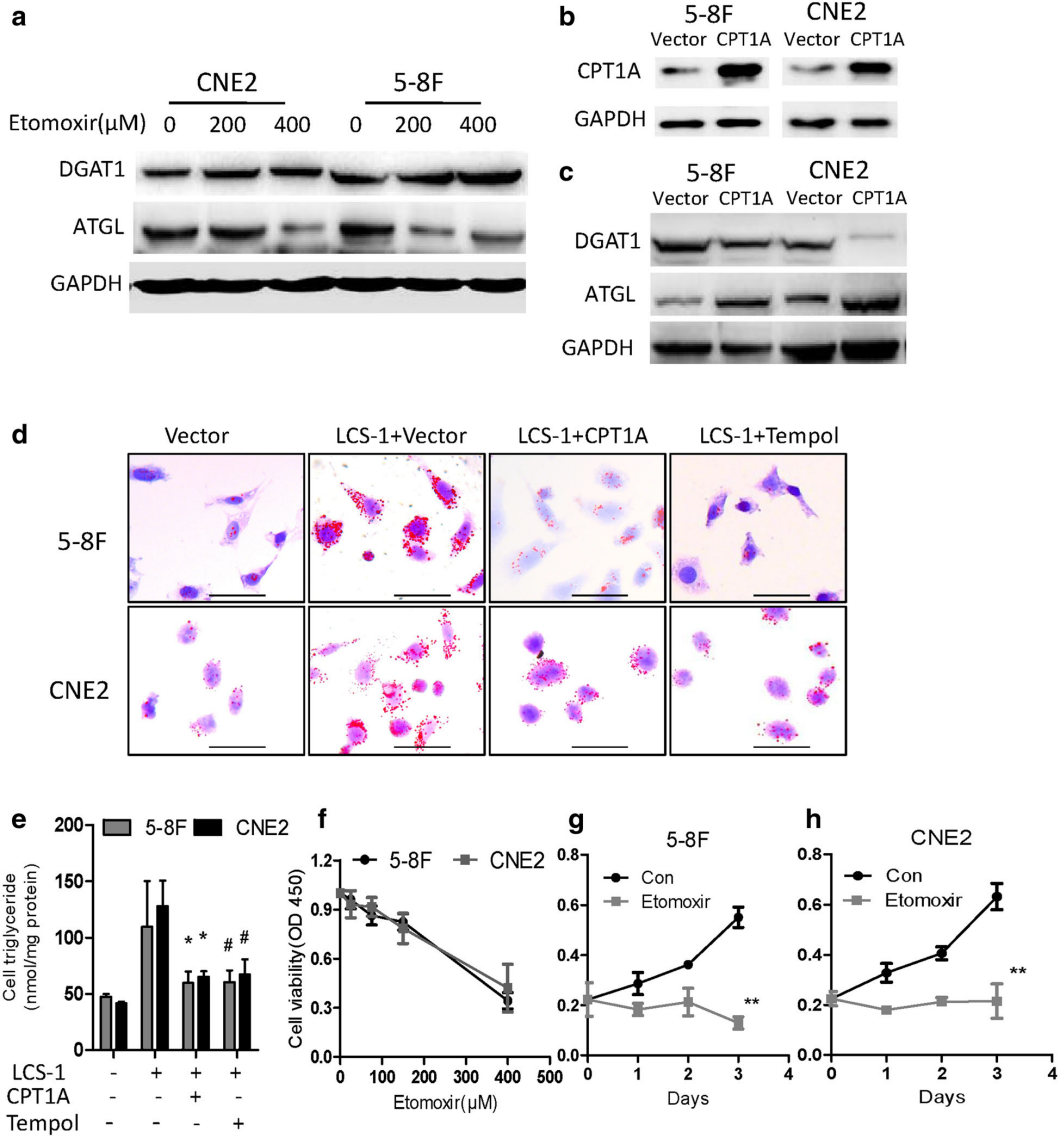
CPT1A suppression induces lipid accumulation and inhibits cell viability. **a** Immunoblotting analysis of ATGL and DGAT1 in 5-8F and CNE2 cells treated with etomoxir at the indicated concentration for 24 h. **b** and **c** Immunoblotting analysis of CPT1A, ATGL and DGAT1 expression in 5-8F and CNE2 cells transfected with CPT1A or vector. **d** Lipid droplet in 5-8F and CNE2 cells were visualized by Oil red O staining and were counterstained with hematoxylin. Scale bar: 50 μm. **e** Detection of intracellular triglyceride content by a triglyceride kit. **f** CCK-8 assays of 5-8F and CNE2 cells treated with etomoxir at the indicated concentration for 24 h. **g** and **h** CCK-8 assays with 5-8F and CNE2 cells treated with etomoxir (400 μM) at the indicated time points. All error bars represent the S.D. of three replicates from two independent experiments. * p < 0.05,**p < 0.01 compared with the control

Then, we further tested the effects of SOD1 on lipid metabolic enzymes expression. Compared with that in the control cells, the CPT1A protein level in the cells treated with LCS-1 or transfected with SOD1 siRNA decreased (Fig. 6b-c). In addition, the overexpression of SOD1 increased CPT1A protein levels (Fig. 6d). In addition, we found that compared with control group in NPC cell, expression of AGPAT1 and DGAT1 at the protein level was upgulated or downregulated in SOD1 overexpression group or SOD1 knockdown group, respectively. The protein level of ATGL was fund remarkably downregulated in SOD1 knockdown group compared with control group in NPC cell (Fig. 6e). As CPT1A is rate-limiting enzyme of the long chain fatty acids transport system controlling entry into the mitochondrion. Recently, it was reported that repress CPT1A enhance fatty acids to lipid droplets for storage [20, 21]. We therefore asked whether SOD1 or CPT1A inhibition induce lipid accumulation. As shown in Fig. 6f and g, inhibiting SOD1 or CPT1A induces lipid accumulation. Further study showed that disruptin CPT1A downregulated DGAT1 and up-regulated ATGL (Fig. 7a), while over-express CPT1A in 5-8F and CNE2 cells reduced protein levels of DGAT1 and up-regulated ATGL protein levels (Fig. 7b-c). CPT1A restoration or TEMPO led to significant decreases lipid accumulation in NPC cells treated with LCS-1(Fig. 7d-e). In addition, etomoxir, a specific CPT1A inhibitor, dose-and time-dependently inhibited the proliferation of 5-8F and CNE2 cells (Fig. 7f-h). To further explore the effect of SOD1 and CPT1A on NPC cells migration and epithelial–mesenchymal transition (EMT). Disrupt SOD1 by LCS-1 or siRNA weaken migration ability of 5-8F and CNE2 cells (Additional file 1: Figure S2) and the expression of twist (mRNA) and vimentin (mRNA and protein) was significantly decreased, coupled with remarkable upregulation of epithelial marker E-cadherin (mRNA and protein) (Additional file 1: Figure S3). However, cell migration was not affected by CPT1A suppression (Additional file 1: Figure S4A-C). Consistently, western blot analysis of the expression of key EMT markers, including E-cadherin and vimentin, showed no significant changes in CPT1A inhibition cells compared with control cells (Additional file 1: Figure S4D-F). These findings suggest that SOD1 promotes the migration of NPC cells independence CPT1A.

In summary, these results suggested that disrupting SOD1 led to the over production of superoxide ion and induced lipid accumulation in NPC cells.

## Discussion

SOD1 is rapidly emerging as a novel target for cancer therapy [22]. As far as we know, this is the first study to demonstrate that SOD1 is a primary regulator of the antioxidant defence system’s ability to counteract superoxide ion production and that this enzyme promotes cell growth in NPC. Compared with those in the normal epithelial cell line NP69 and NETs, SOD1 expression levels in a panel of NPC cells and tumour tissues respectively increased (Fig. 1). Previous studies have shown that during transformation, breast cancer cells predominately expressing SOD2 to expressing SOD1 as the primary superoxide dismutase isoform [23]. In absence of SOD2, superoxide levels are elevated and may cause irreversible damage. For this reason, SOD1 must maintain the viability of cancer cells. Therefore, clarifying the function and underlying mechanism of SOD1 in nasopharyngeal carcinoma development has become a core issue in the treatment of NPC.

In this study, we first investigated how the disruption of SOD1 affects the antioxidant defence system and nasopharyngeal carcinoma cell growth. Disrupting SOD1 by LCS-1 treatment or knocking down SOD1 drastically reduced cell viability, and the overexpression SOD1 decreased cellular susceptibility to oxidative stress in vitro and in vivo (Fig. 2-4). Previous studies have shown that inhibiting SOD1 either by shRNA or the SOD1 inhibitor ATN-224 drastically reduces the ability of the lung carcinoma cell line A549 to form colonies on soft agar [24]. LCS-1 and SOD1 siRNAs inhibit the growth of LCS-1-sensitive lung adenocarcinoma cell lines [25]. In addition, in leukaemia, Huang et al. identified SOD1 as a target of an anti-cancer agent [26]. These data support the hypothesis that SOD1 may be essential for the adaptation of cancer cells to increased oxidative stress.

Altered redox status is a key biochemical feature that is frequently observed in tumour [1]. ROS such as superoxide and hydrogen peroxide have been implicated in the development and progression of tumours. Under normal physiological conditions, ROS participate in redox reactions and serve as second messengers for regulatory functions. ROS are important for cell survival and tumourigenesis, but large increases in ROS levels usually cause cell death, thus requiring a robustly active antioxidant system to prevent cellular damage [7, 27].

Our results indicate that cellular O_2_•- is important for LCS-1-induced cytotoxicity. SODs are the major antioxidant defence systems against O_2_•-, and three isoforms of SOD exist in mammals: cytoplasmic SOD1 (Cu/ZnSOD), mitochondrial SOD2 (MnSOD) and extracellular SOD3 (Cu/ZnSOD) [4].The increased O_2_•- level induced by SOD1 inhibition during cell apoptosis can be reversed by the superoxide scavenger TEMPO (Fig. 5). Recently, studies have shown that SOD1 not only functions as an antioxidant enzyme but also plays a key role in cellular metabolism. Compared with normal cells, cancer cells greatly limit pyruvate flux into mitochondrial oxidative phosphorylation and upregulate FA oxidation to support tumour growth [15, 28]. In the context of cancer metabolism, FA oxidation has unveiled new and exciting therapeutic opportunities, to evaluate if SOD1 affects NPC cell metabolism, the key enzymes involved in mitochondrial FA metabolism were examined. Our findings demonstrated that SOD1 mainly improves the growth performance of NPC cells via CPT1A-mediated lipid metabolism (Fig. 6). Moreover, CPT1A downregulation has been observed in SOD1 G93A cells [29]. Targeting CPT1A could be a beneficial regimen to improve the therapeutic effects of radiotherapy in NPC patients [21]. Future studies should focus on the development of more selective SOD1 inhibitors, their combinatorial effect of these inhibitors with lipid metabolism drugs and the mechanism behind these effects.

## Conclusions

In conclusion, understanding the precise roles of SOD1 in advanced NPC may enable it to be used as a prognostic biomarker and may aid in the development of novel therapeutic strategies.

## Additional file

Additional file 1: **Table S1.** Primers sequence for qPCR, **Figure S1.** Effects of PDH on cell viability of NPC cells, **Figure S2.** Effects of SOD1 on cell migration of NPC cells. **Figure S3.** Effects of SOD1 on EMT makers of NPC cells, **Figure S4.** Effects of Etomoxir on cell migration of NPC cells. (PDF 389 kb)

## Abbreviations

APGAT1: 1-acylglycerol-3-phosphate O-acyltransferase 1
ATGL: Adipose triglyceride lipase
CPT1A: Carnitine palmitoyltransferase 1A
DGAT1: Diacylglycerol O-acyltransferase 1
EMT: Epithelial-to-mesenchymal transition
FAO: Fatty acid oxidation
NPC: Nasopharyngeal carcinoma
ROS: Reactive oxygen species
SOD: Superoxide dismutase
