# Supplementary material for: Disrupting SOD1 activity inhibits cell growth and enhances lipid accumulation in nasopharyngeal carcinoma
Source: Cell Commun Signal. 2018 Jun 11;16:28. doi: 10.1186/s12964-018-0240-3 (PMC5996554; doi:10.1186/s12964-018-0240-3)
Supplement: Supplementary file 1 — Table S1. Primers sequence for qPCR, Figure S1. Effects of PDH on cell viability of NPC cells, Figure S2. Effects of SOD1 on cell migration of NPC cells. Figure S3. Effects of SOD1 on EMT makers of NPC cells, Figure S4. Effects of Etomoxir on cell migration of NPC cells. (PDF 389 kb) [file 12964_2018_240_MOESM1_ESM.pdf]

Table S1: Primers sequence for qPCR

Gene	Sequence (5'-3')
SOD1	TGGTTTGCGTCGTAGTCTCC
	CTTCGTCGCCATAACTCGCT
β-Actin	GCACTCTTCCAGCTTCCTT
	GTTGGCGTACAGGTCTTTGC
PDHE1A	TTCTCAGAAGCCGGCAAGC
	AGCACTGTTGTGACAGGAGG
CPT1A	CCTCCAGTTGGCTTATCGTG
	TTCTTCGTCTGGCTGGACAT
FASN	AAGGACCTGTCTAGGTTTGATGC
	TGGCTTCATAGGTGACTTCCA
PDK1	TCACAGAGGAGCGTTTCTGG
	TCACTGATCCTTCCTGCTTCC
ACLY	AGTCCAGGGCCGATGAGGTGG
	GAGGGTGGTGCTCTTTCCTTGCAG
MCAD	TGGATAACCAACGGAGGAAAAG
	CTGGGGTATCTGCTTCCACA
E-cadherin	ATTTTTCCCTCGACACCCGAT
	TCCCAGGCGTAGACCAAGA
Vimentin	CGGGAGAAATTGCAGGAGGA
	AAGGTCAAGACGTGCCAGAG
Twist	GTCCGCAGTCTTACGAGGAG
	GCTTGAGGGTCTGAATCTTGCT

Supplementary Figure 1

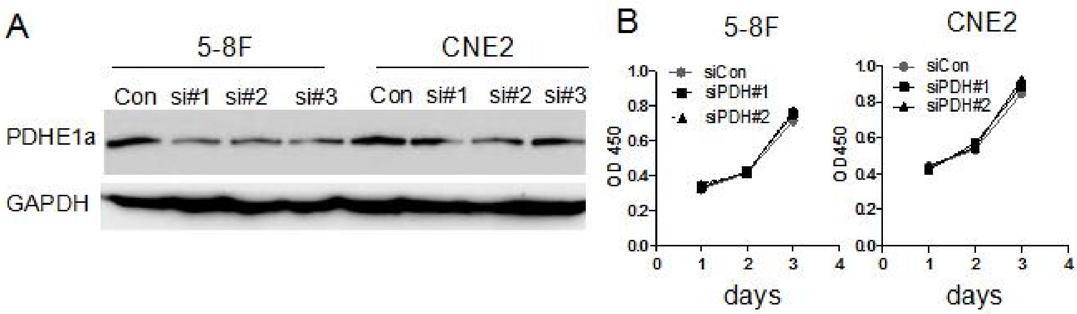

Fig.S1: **Effects of PDH on cell viability of NPC cells.** **A.** Western blot analysis of pyruvate dehydrogenase subunit E1A in 5-8F and CNE2 cells transfected with three specific siRNAs or control siRNA for 24h. **B.** Cell viability in 5-8F and CNE2 cells with PDHE1A knocked down.

Supplementary Figure 2

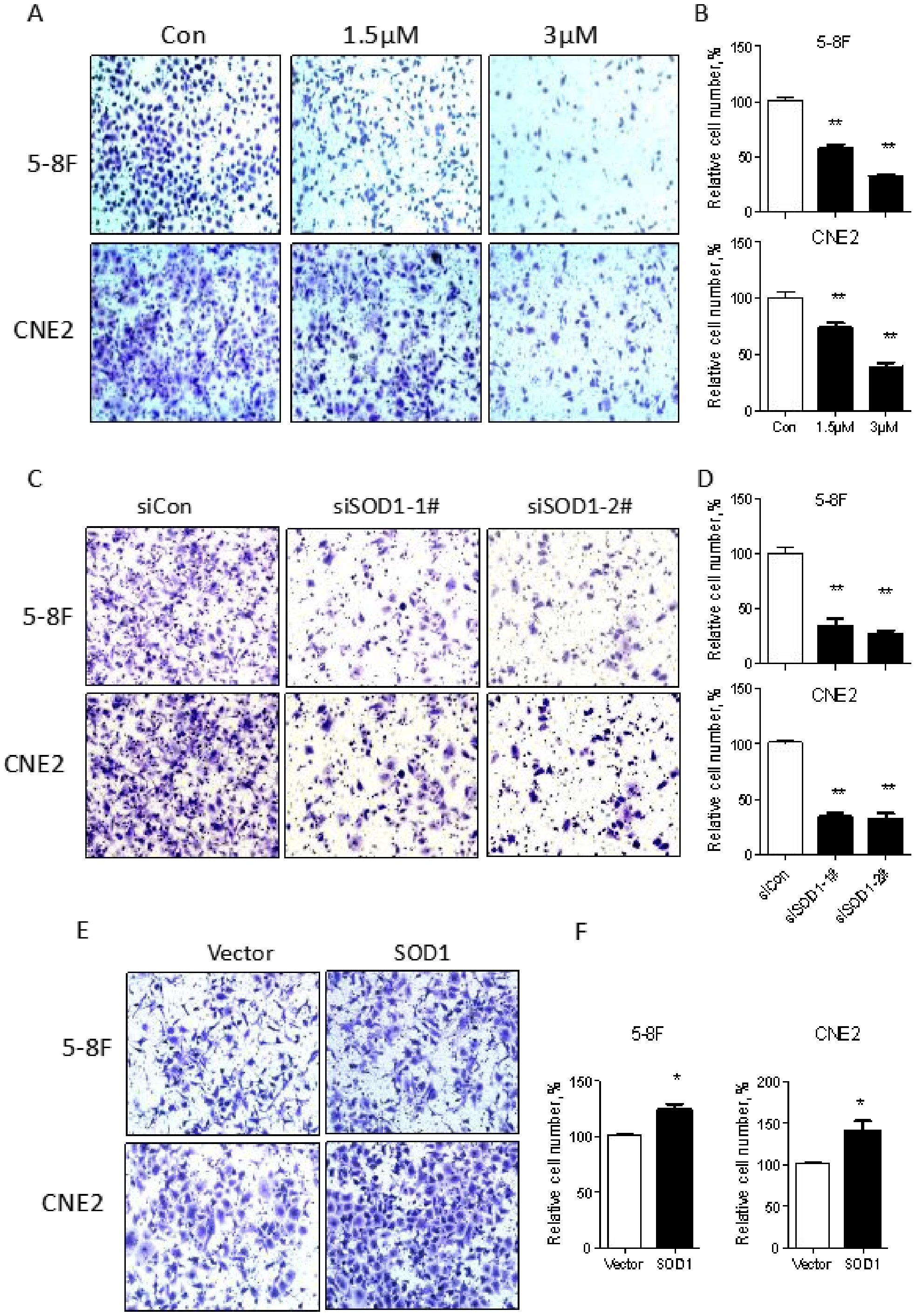

Fig.S2: **Effects of SOD1 on cell migration of NPC cells. A&B.** Migration ability of 5-8F and CNE2 cells was remarkably downregulated in LCS-1 groups compared with control group. **C&D.** Migration ability of 5-8F and CNE2 cells was remarkably downregulated in SOD1 knockdown groups compared with control group. **E&F.** Elevated SOD1 levels increased migration ability of 5-8F and CNE2 cells. All error bars represent the S.D. of three replicates from two independent experiments. *p<0.05,**p<0.01.

Supplementary Figure 3

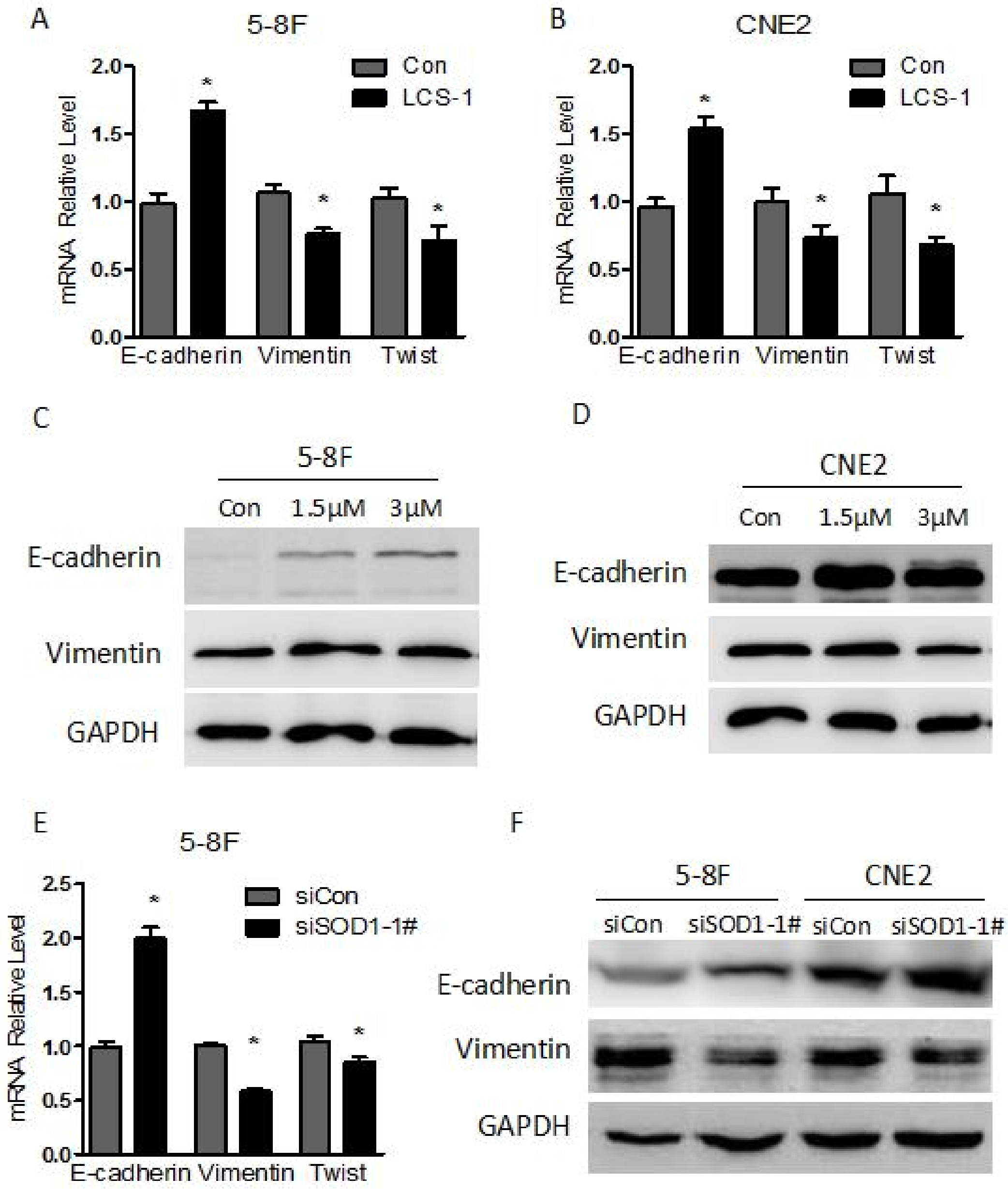

Fig.S3: **Effects of SOD1 on EMT makers of NPC cells.** Expression of vimentin and twist at the mRNA level was remarkably downregulated and the mRNA level of E-cadherin was upregulated in LCS-1 groups compared with control group in 5-8F(**A**) and CNE2 (**B**) cell for 24h. **C&D.** Immunoblotting analysis of E-cadherin and vimentin expression in 5-8F and CNE2 cells treated with LCS-1 for 24h. **E.** Expression of vimentin and twist at the mRNA level was remarkably downregulated and the mRNA level of E-cadherin was upregulated in SOD1 kncokdown group compared with control group in 5-8F cell. **F.** Immunoblotting analysis of E-cadherin and vimentin expression in 5-8F and CNE2 cells treated with SOD1 siRNA for 24h. All error bars represent the S.D. of three replicates from two independent experiments.;*p<0.05

Supplementary Figure 4

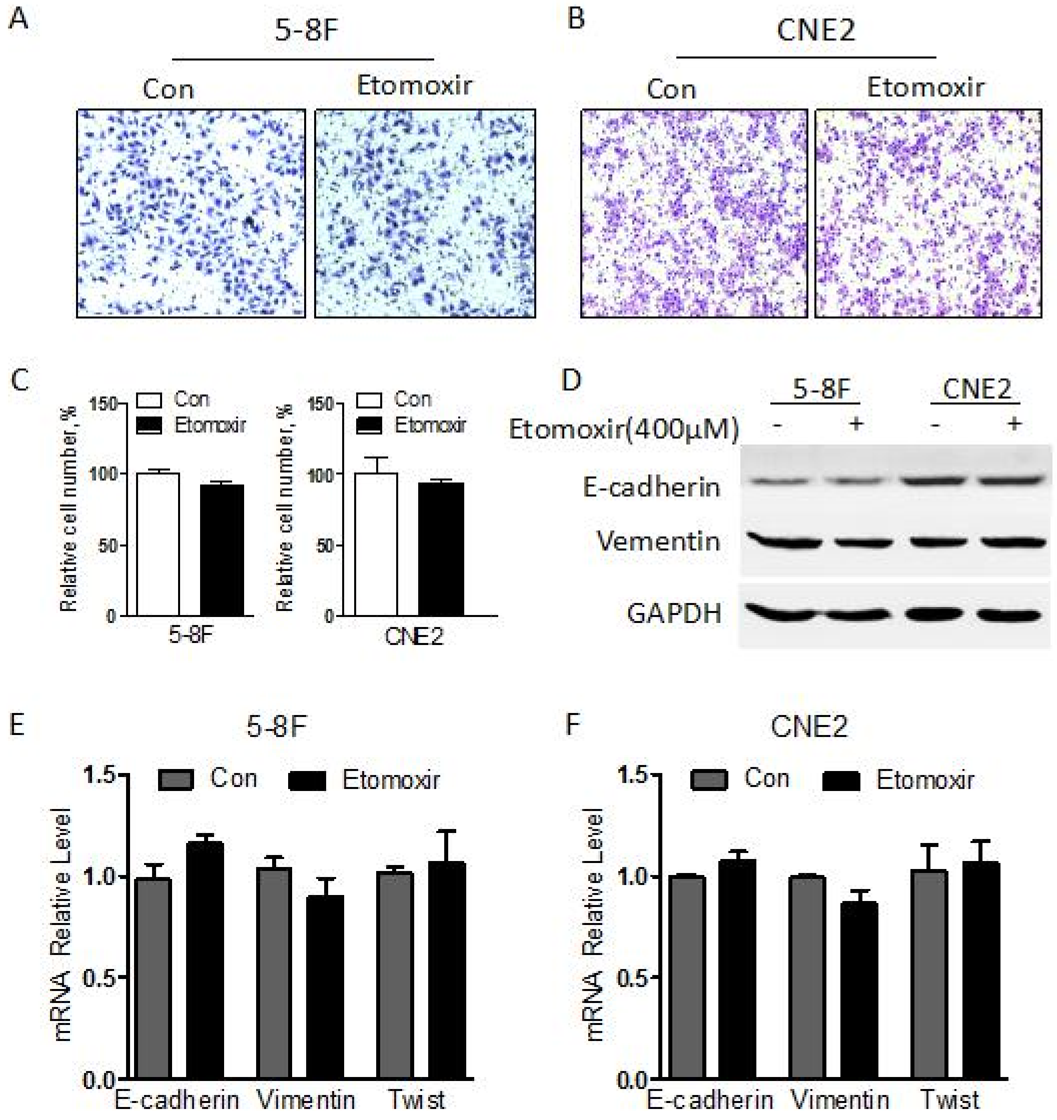

Fig.S4: **Effects of Etomoxir on cell migration of NPC cells.** Migration ability of 5-8F (**A**) and CNE2 cells (**B**) was shown in etomoxir group compared with control group for 24h. **C.** Quantification of the number of migration of NPC cells. **D.** western blot analysis of E-cadherin and vimentin expression in 5-8F and CNE2 cells treated with 400 μM etomoxir for 24h. **E&F.** Expression of E-cadherin， vimentin and twist at the mRNA level in 5-8F(**A**) and CNE2 (**B**) cell for 24h.
